# Direct Oral Anticoagulants in Old and Frail Patients with Atrial Fibrillation: The Advantages of an Anticoagulation Service

**DOI:** 10.3390/jpm12081339

**Published:** 2022-08-20

**Authors:** Maria Cristina Vedovati, Giancarlo Agnelli

**Affiliations:** 1 Internal Vascular and Emergency Medicine—Stroke Unit, University of Perugia, 06129 Perugia, Italy; 2 IRCCS Istituti Scientifici Maugeri, 27100 Pavia, Italy

**Keywords:** atrial fibrillation, direct oral anticoagulants, frail patients

## Abstract

Despite the recent advancements, oral anticoagulation is still challenging in some patients and this is the case for old and frail patients. The large majority of frail patients with atrial fibrillation should receive anticoagulation since the associated benefits outweigh the risk of bleeding. A multidisciplinary consensus document on the use and prescription of direct oral anticoagulants (DOACs) in older and frail patients with atrial fibrillation has been recently published. In this manuscript we provide a comment on this document and add insights into the management of these patients. The new DOAC age had imposed a paradigm shift in the management of patients with the need for clinically-oriented services rather than laboratory-oriented services. In this paper we provide tools for a structured patient-oriented DOACs treatment service supported by a multidisciplinary approach.

## 1. Introduction

The advent of direct oral anticoagulants (DOACs) has been associated with one of the most substantial changes in cardiovascular therapy in the last decades. DOACs (apixaban, dabigatran, edoxaban, and rivaroxaban) are now considered as the first choice for the prevention of stroke in patients with non-valvular atrial fibrillation (AF) and for the treatment of venous thromboembolism and have largely replaced vitamin K antagonists (VKA) (warfarin, acenocoumarol, phenprocoumon). This switch is essentially based on the results of large clinical trials with DOACs and related to the predictability of their dose–effect relationship, their administration at fixed doses with no need for laboratory monitoring, their relatively modest interaction with drugs and foods, and, overall, their excellent safety profile [1].

Data from nine databases (claims or electronic health records) in the United States and Europe, including more than three million patients with AF prescribed for the first time with an oral anticoagulant, showed that the prescription of VKA has significantly declined from 2010 to 2017 while the prescription of DOACs has significantly increased [2]. Indeed, in 2010, more than 87.5% of patients started their anticoagulant treatment with VKAs, while already in 2017, the majority of patients started with DOACs. Indeed, in 2017, less than 5 years after their introduction in clinical practice, the prevalent use of DOACs was 87.0% in the United States, 88.3% in Belgium, 93.1% in France, 88.4% in Germany, and 86.1–86.7% in the United Kingdom.

Although data from the literature indicate a good risk-to-benefit ratio for DOACs in the majority of patients with non-valvular AF, clinicians are still reluctant to prescribe these agents in some patient categories that were less represented in phase 3 randomized registration trials. Patients with active cancer, renal impairment, and those who are old or very old and frail are among these patients [3,4,5,6]. Frailty is a multidimensional syndrome of poor physiological reserve leading to increased vulnerability to stressors, resulting in dependency and poor health outcomes [7].

In a recently published retrospective study, among 75,796 patients discharged from an emergency department or hospital between 2009 and 2019 with a new diagnosis of non-valvular AF and an indication for anticoagulant treatment, 22.6% of patients were categorized as frail [7]. The Hospital Frailty Risk Score was used to define frailty, and the CHA2DS2-VASc and CHADS-65 scores were used to assess if anticoagulation was indicated. The study showed that, although guideline criteria for anticoagulation were more commonly met by frail patients than non-frail patients (92.1% vs. 74.2% for CHA2DS2-VASc and 96.8% vs. 85.8% for CHADS-65; both *p* < 0.001), frail patients were less likely to receive anticoagulant treatment, even after those with contraindications to anticoagulation were excluded (adjusted odds ratio (OR) 0.61, 95% confidence interval (CI) 0.58–0.64). In this study, the introduction of DOACs was associated with increased anticoagulation rates in frail patients without bridging the gap with not frail patients. Indeed, the rate of anticoagulant prescription increased more in non-frail patients (from 42.4% to 68.2%) than in frail patients (from 29.0% to 52.2%), and frail patients were less likely to receive DOACs than warfarin (adjusted OR 0.66, 95% CI 0.54–0.81). Of note, the most recently released practical guide on the use of DOACs from the European Heart Rhythm Association (EHRA) 2021 clearly states that frailty should not be considered as a reason to preclude patients from the benefit of oral anticoagulants and DOACs in particular [8].

This statement of EHRA is supported by solid data, including a recent patient-level meta-analysis [9]. In this analysis, the relationship between age, as a continuous variable, and outcomes were explored using data from the four RCTs that compared DOACs with warfarin. This analysis showed that the treatment effects for standard- and lower-dose DOACs in comparison to VKAs were consistent across age and sex for the outcomes of stroke, systemic embolism, and death. Actually, a trend in favor of DOACs in comparison to VKAs was shown as age increased. On the other hand, a reduction in major bleedings with DOACs in comparison to VKAs was observed only in young patients. Data in frail patients are available from an RCT trial performed in Japan [10]. The ELDERCARE-AF trial categorized patients at randomization as robust, prefrail, or frail using a standardized frailty assessment tool [10]. The results of this study showed that edoxaban (15 mg daily) was superior to the placebo in preventing stroke or systemic embolism (HR 0.35, 95% CI 0.14–0.87) and was not associated with a significantly higher incidence of major bleeding in comparison to placebo (HR 1.67, 95% CI 0.58–4.75).

The multidisciplinary consensus documents published by Proietti et al. in this issue of the *Journal of Personalized Medicine* specifically addresses conditions with relatively limited evidence on the use of DOACs such as multimorbidity, polypharmacy, high risk for falling, dementia, frailty, or older age [11]. The focus is mainly placed on frailty, which has a variable prevalence, probably due to the different definitions used in the related reports and because, regardless of the definition, it has an influence on the risk for adverse outcomes, in particular on bleeding [12].

Proietti et al. propose an integrated clinical approach involving a multidisciplinary team to face the clinical aspects related to AF in old and frail patients and the use of geriatric comprehensive assessment (GCA) [11]. Geriatric comprehensive assessment is usually defined as “a multidimensional, multidisciplinary process which identifies medical, social and functional needs and the development of an integrated/coordinated care plan to meet those needs”. It has been demonstrated that CGA can be performed in a primary care context and significantly reduce the need for hospital care [13].

Comprehensive care is a crucial issue for the management of patients with a chronic disease requiring long-term treatment and follow-up, such as patients with AF. The management of these patients should provide an integrated approach between general practitioner and hospital facilities dedicated to the management of anticoagulant therapy.

To complement the comprehensive care proposed by Proietti et al. [11], we would like to focus on the importance of adequate services for the management of the anticoagulant treatment in old and frail patients.

VKA anticoagulant clinics were set up to address some limitations of warfarin, including the need for laboratory monitoring and dose adjustment, and have been a successful model of care for several years. Indeed, in addition to laboratory monitoring, anticoagulation clinics provide VKA-treated patients with clinical surveillance and counselling. However, the new DOAC age had imposed a paradigm shift in the management of patients with the need for clinically oriented services rather than laboratory-oriented services. Due to the absence of laboratory tests to assess the level of anticoagulation, treatment with DOAC is more feasible, but it should not be considered trivial as it requires careful clinical judgment for the balance between clinical benefits and risks for treatment-emergent bleeding. Nowadays, the majority of anticoagulated patients are not taken care of by the traditional “anticoagulation clinics”, as they are treated with DOACs and, therefore, do not require laboratory monitoring. As a consequence, DOACs-treated patients run the risk of being missed for follow-up by clinicians with expertise on anticoagulant treatment. For these reasons, it would be appropriate to organize a clinical service able to follow patients treated with DOACs, and this is particularly crucial for old and frail patients.

In this view, a structured outpatient service focused on the treatment with DOACs should provide patients with start-up and follow-up procedures across a number of actions.

The start-up procedures should include:

(i) Assessment of the indication for anticoagulant treatment. This requires the estimation of the risks and benefits of DOACs treatment in an individual patient. The identification of the proper DOAC and its dose would avoid an inappropriate prescription and over and under-dosing. Baseline laboratory exams should be checked, as anemia and renal insufficiency are risk factors for bleeding during anticoagulant treatment and could promote to the use of reduced doses of DOACs in fragile patients.
(ii) Provide education to the patient. Patient education tools are important to improve strict adherence to therapy and to avoid insufficient patient attention to new drugs.
(iii) Hands-out anticoagulation card. DOACs do not alter the results of standard coagulation tests in a dose-dependent manner. In emergency situations, the availability of a hands-out anticoagulation card can enable physicians to rapidly be informed on patients’ exposure to anticoagulant agents. This information can be crucial in case reversal of anticoagulation is required.
(iv) Organize the follow-up program. The patients should receive scheduled follow-up data and the appropriate timing for laboratory controls or any specialistic visits.

The follow-up procedures should include:

(i) Inquire about the occurrence of bleeding and thromboembolic events.
(ii) Learn about co-medication, including over-the-counter medications.
(iii) Verify patient adherence to treatment.

This service is particularly useful for the management of patients with challenging situations (such as old and frail patients) and the support for patients, general practitioners, and other specialists when dealing with specific situations. These include the clinical management of both major and minor bleeding complications (needs for DOACs withdrawal, needs for reversal agents), managing the switching from VKAs, and counselling for invasive procedure. The management of bleeding complications in patients treated with DOACs is only partially similar to that of patients treated with VKAs. This service may offer a support for the implementation of knowledge concerning the reversal process in case of DOACs-induced bleeding complications and for the management of emergency situations. The administration of prothrombin complex concentrates or fresh frozen plasma has been reported to have partial effects on reversing the anticoagulant effect of DOACs. In addition, specific antidotes for both anti-Xa and anti-II inhibitors are indicated in case of life-threatening situations. In case of invasive procedures, patients could require temporary discontinuation of DOACs to reduce the peri-procedural risk of bleeding. This occurrence is quite common since up to 25% of patients with AF enrolled in clinical trials with DOACs required temporary discontinuation of anticoagulation for procedures or surgical intervention. Thus, experts of the DOAC service may provide a call service to support physicians and patients to optimize the clinical management of both minor and major bleeding complications and on interventional procedures. Indeed, most of the clinical situations where a structured service for DOAC management could be of help are particularly common in old and frail patients, and thus this service should be integrated in the organization model proposed by Proietti et al. [12].

In conclusion, despite the recent advancements, oral anticoagulation is still challenging in some patients, and this is the case for old and frail patients. The large majority of frail patients with AF should receive anticoagulation since the associated benefits outweigh the risk of bleeding. An individualized patient-centered approach should be supported by a multidisciplinary approach and is recommended for those who are older and frail. A structured patient-oriented DOACs treatment service could facilitate the management of anticoagulation in these patients.

## Data Availability

Not applicable.

## References

[1] Hindricks G., Potpara T., Dagres N., Arbelo E., Bax J.J., Blomström-Lundqvist C., Boriani G., Castella M., Dan G.A., Dilaveris P.E. (2021). 2020 ESC Guidelines for the diagnosis and management of atrial fibrillation developed in collaboration with the European Association for Cardio-Thoracic Surgery (EACTS): The Task Force for the diagnosis and management of atrial fibrillation of the European Society of Cardiology (ESC) Developed with the special contribution of the European Heart Rhythm Association (EHRA) of the ESC. Eur. Heart J..

[2] Vora P., Morgan Stewart H., Russell B., Asiimwe A., Brobert G. (2022). Time Trends and Treatment Pathways in Prescribing Individual Oral Anticoagulants in Patients with Nonvalvular Atrial Fibrillation: An Observational Study of More than Three Million Patients from Europe and the United States. Int. J. Clin. Pract..

[3] Giustozzi M., Vedovati M.C., Verso M., Scrucca L., Conti S., Verdecchia P., Bogliari G., Pierpaoli L., Agnelli G., Becattini C. (2019). Patients aged 90 years or older with atrial fibrillation treated with oral anticoagulants: A multicentre observational study. Int. J. Cardiol..

[4] Becattini C., Giustozzi M., Ranalli M.G., Bogliari G., Cianella F., Verso M., Agnelli G., Vedovati M.C. (2018). Variation of renal function over time is associated with major bleeding in patients treated with direct oral anticoagulants for atrial fibrillation. J. Thromb. Haemost..

[5] Vedovati M.C., Giustozzi M., Verdecchia P., Pierpaoli L., Conti S., Verso M., Di Filippo F., Marchesini E., Bogliari G., Agnelli G. (2018). Patients with cancer and atrial fibrillation treated with doacs: A prospective cohort study. Int. J. Cardiol..

[6] De Simone V., Mugnolo A., Zanotto G., Morando G. (2020). Direct oral anticoagulants for patients aged over 80 years in nonvalvular atrial fibrillation: The impact of frailty. J. Cardiovasc. Med..

[7] Orlandi M., Dover D.C., Sandhu R.K., Hawkins N.M., Kaul P., McAlister F.A. (2022). The Introduction of Direct Oral Anticoagulants Has Not Resolved Treatment Gaps for Frail Patients with Nonvalvular Atrial Fibrillation. Can. J. Cardiol..

[8] Steffel J., Collins R., Antz M., Cornu P., Desteghe L., Haeusler K.G., Oldgren J., Reinecke H., Roldan-Schilling V., Rowell N. (2021). 2021 European Heart Rhythm Association Practical Guide on the Use of Non-Vitamin K Antagonist Oral Anticoagulants in Patients with Atrial Fibrillation. EP Eur..

[9] Caldeira D., Nunes-Ferreira A., Rodrigues R., Vicente E., Pinto F.J., Ferreira J.J. (2019). Non-vitamin K antagonist oral anticoagulants in elderly patients with atrial fibrillation: A systematic review with meta-analysis and trial sequential analysis. Arch. Gerontol. Geriatr..

[10] Okumura K., Akao M., Yoshida T., Kawata M., Okazaki O., Akashi S., Eshima K., Tanizawa K., Fukuzawa M., Hayashi T. (2020). Low-Dose Edoxaban in Very Elderly Patients with Atrial Fibrillation. N. Engl. J. Med..

[11] Proietti M., Camera M., Gallieni M., Gianturco L., Gidaro A., Piemontese C., Pizzetti G., Redaelli F., Scimeca B., Tadeo C.S. (2022). Use and Prescription of Direct Oral Anticoagulants in Older and Frail Patients with Atrial Fibrillation: A Multidisciplinary Consensus Document. J. Pers. Med..

[12] Proietti M., Romiti G.F., Raparelli V., Diemberger I., Boriani G., Vecchia L.A.D., Bellelli G., Marzetti E., Lip G.Y., Cesari M. (2022). Frailty Prevalence and Impact on Outcomes in Patients with Atrial Fibrillation: A Systematic Review and Meta-Analysis of 1,187,000 Patients. Ageing Res. Rev..

[13] Sletvold O., Helbostad J.L., Thingstad P., Taraldsen K., Prestmo A., Lamb S.E., Aamodt A., Johnsen R., Magnussen J., Saltvedt I. (2021). Costs and effects of comprehensive geriatric assessment in primary care for older adults with high risk for hospitalisation. BMC Geriatr..

